# Effectiveness of lifestyle-modification approach (a randomized-controlled program of diet restriction and treadmill walking exercise) on elderly’s metabolic syndrome-associated subjective tinnitus

**DOI:** 10.1007/s00405-025-09494-7

**Published:** 2025-06-11

**Authors:** Ali Mohamed Ali Ismail, Ahmed Mahmoud Nasr Tolba

**Affiliations:** 1 Department of Physical Therapy for Cardiovascular/Respiratory Disorder and Geriatrics, Faculty of Physical Therapy, Cairo University, Giza, Egypt; 2 Basic Science Department, Faculty of Physical Therapy, Delta University for Science and Technology, International Coastal Road, Gamassa, Dakahlia, Egypt

**Keywords:** Diet, Exercise, Tinnitus, Metabolic syndrome, Elderly

## Abstract

**Introduction:**

Chronic subjective tinnitus complaint (CSTC) is highly prevalent in metabolic syndrome (MS) elderly. In 60 obese MS elderly with CSTC, studying the response of CSTC-related discomfort, MS components, and CSTC severity to lifestyle-modification approach was the aim of this study.

**Methods:**

In this randomized/controlled lifestyle-modification trial, the recruitment of 60 obese MS elderly with CSTC from a local general hospital was randomly executed. The recruited elderly with MS and CSTC were randomly assigned to a study group (performed lifestyle-modification approach and the components of this 12-week approach were on-electrical treadmill walking plus diet restriction) or waitlist/control group (*n* = 30). Besides MS components/variables such as elderly’s waist circumference (WC), elderly’s fasting glucose in serum (FGIS), elderly’s triglycerides (TriGly), elderly’s blood pressure, and elderly’s high-density lipoprotein (HDLipo), lifestyle-modification approach’s outcomes of this trial were elderly’s body mass index, visual analogue scale (VAS) of CSTC-related discomfort, tinnitus handicap inventory (THI), and VAS of CSTC severity.

**Results:**

Tracking all outcomes of this lifestyle-modification approach in the study group (*n* = 30 MS elderly with CSTC) showed significant improvements while the control group’s outcome did not improve.

**Conclusion:**

BMI, VAS of CSTC severity, components of MS (WC, FGIS, blood pressure, TriGly, and HDLipo), THI, and VAS of CSTC-related discomfort could be improved after involving obese MS elderly with CSTS in a 12-week lifestyle-modification approach.

**Trial registration number:**

NCT06702085.

## Introduction

Thirteen to seventeen percent of people experience tinnitus. This number includes 33% of the senior population [1]. The most typical type of tinnitus complaint, particularly in older persons, is subjective tinnitus. The term “chronic subjective tinnitus complaint” (CSTC) refers to an aural/audible sensation (noise, hissing, ringing, and/or buzzing) in the ear that is consciously perceived over a period of time longer than six months and is unrelated to external stimuli. Only patients can experience CSTC because doctors cannot hear the by-patient-reported complaints (noise, hissing, ringing, and/or buzzing) and detect no objective signs of ear pathology [2].

Although CSTC is frequently associated with various auditory troubles for the elderly, it may also be a sign of other health issues/problems, such as cardiovascular risk factors or diseases [3, 4]. Obesity (especially the central form, known as apple-shaped obesity), dyslipoproteinaemia (DL) with atherosclerosis/arteriosclerosis, arterial hypertension (AH), and diabetes mellitus (DM) or insulin resistance (IR)– as defining components of metabolic syndrome (MS) [5–7] **-** are significant and frequent causes of CSTC [3].

The prevalence of CATC in MS patients may be explained by MS-related variables/components. For the first component of MS, obesity, according to Gallus et al. [8], those with a body mass index (BMI) > 30 kg/m^2^ had a higher prevalence of CSTC than people with a normal BMI (i.e. 30 kg/m^2^). Obesity has been identified as a causative/inducing factor for CSTC along with its significantly linked elevated levels of oxidative stress (OS) and chronic systemic inflammation [9].

Regarding the second component of MS, AH, the mechanism behind the association of AH and tinnitus is not well known. According to animal studies/experiments, AH may trigger the development of present tinnitus complaints or exacerbate previous tinnitus complaints by two main processes/mechanisms, high blood pressure may negatively impact the cochlear microcirculation, and many antihypertensive pharmacotherapies may cause local toxicity of ear tissues [10].

Regarding the third component of MS, diabetes mellitus or IR, the mechanism behind the association of IR, DM, and tinnitus is sophisticated. Like the brain, the inner ear’s functions are dependent on levels of energy reserves. The circulating levels of blood oxygen and glucose are directly responsible for the inner ear’s metabolism and functions. Changes/alternations in glucose metabolism have a high potential to impair the inner ear’s functionality [3]. Also, DM-induced complications such as local endothelial dysfunction, cochlear microangiopathy, the elevation of OS and chronic systemic inflammation, and auditory neuropathy have great potential for disturbing the inner ear’s functions, hence tinnitus may appear [11].

Regarding the fourth component of MS, DL, the mechanism behind the association of DL and CSTC is documented. The possible contributors or mechanisms for DL-induced CSTC include a buildup of lipoproteins in the ear’s arterial walls, compromised cochlear and ear microcirculation, intensified local OS, chronic arteriosclerotic and vasoconstricting changes of ear’s blood vessels, and impaired cochlear metabolism/oxygenation [12].

Because the available therapies for CSTC are so ineffective, both patients and otorhinolaryngology doctors frequently experience frustration with this medical condition/problem. Many people with CSTC are particularly receptive to and favor the use of complementary/alternative therapies [2]. Tinnitus retraining protocols, counseling, cognitive and behavioral therapies, and auditory/tinnitus sound therapy can be very expensive and have varying degrees of success/efficacy [13].

A recent non-expensive complementary/alternative approach utilizing diet restriction with increased exertion levels was recommended as a treatment of obesity-associated CSTC. This approach was associated with an improvement in tinnitus symptoms and CSTC-related quality of life (QoL) [14, 15].

Investigating the effect of this approach on CSTC in the elderly with MS components (obesity, DL, IR and/or DM, and AH) was not explored before. This paper’s aim was the first of its type to explore the effect of a lifestyle-modification intervention (12 weeks of diet restriction with increased exertion levels) on CSTC in elderly with MS.

## Materials and methods

### Design

The identifier of this lifestyle-modification approach is NCT06702085. In patients with MS and CSTC, authors employed a randomized controlled lifestyle-modification approach during the period from 1st January 2024 to 1st January 2025.

### Settings

Patients with MS and CSTC were recruited from Mit Ghamr (Dakadous) general hospital.

### Ethics of this CSTC study

Besides Helsinki guidelines/ethics in lifestyle-modification trials, consenting patients with MS and CSTC were applied. To apply the lifestyle-modification approach in patients with MS and CSTC, local institutional clearance (P.T.REC/012/004623) was obtained.

### Subjects

In this lifestyle-modification trial, CSTC patients with class-I obesity were included. Over a period of time longer than six months, MS patients (*n* = 60) aged ≥ 65 years old had bilateral CSTC.

Three or more indicators defined MS in the older participants: (i) women’s waist circumference (WC) ≥ 88 cm or men’s WC ≥ 102 cm, (ii) receiving pharmacological therapies for AH or having AR ≥ 130/85 mmHg, (iii) having at least one component of DL (i.e. men’s low high-density lipoprotein (HDLipo) < 40 mg/dl, women’s HDLipo < 50 mg/dl, and/or elderly’s triglycerides (TriGly) ≥ 150 mg/dl, (IV) having IR (i.e. elderly’s fasting glucose in serum (FGIS) ≥ 110 mg/dl [5].

Metabolic-syndrome elderly with liver or kidney diseases, by-physician reported previous or recently diagnosed local ear diseases and/or trauma, cardiorespiratory disorders, ear tumors, joint problems in lower limbs, alcohol or illegal drug consumption, unstable psychological status, partial/complete hearing loss, and neurologic/cerebral disorder were ruled out.

### Randomization

**Fig. 1:**
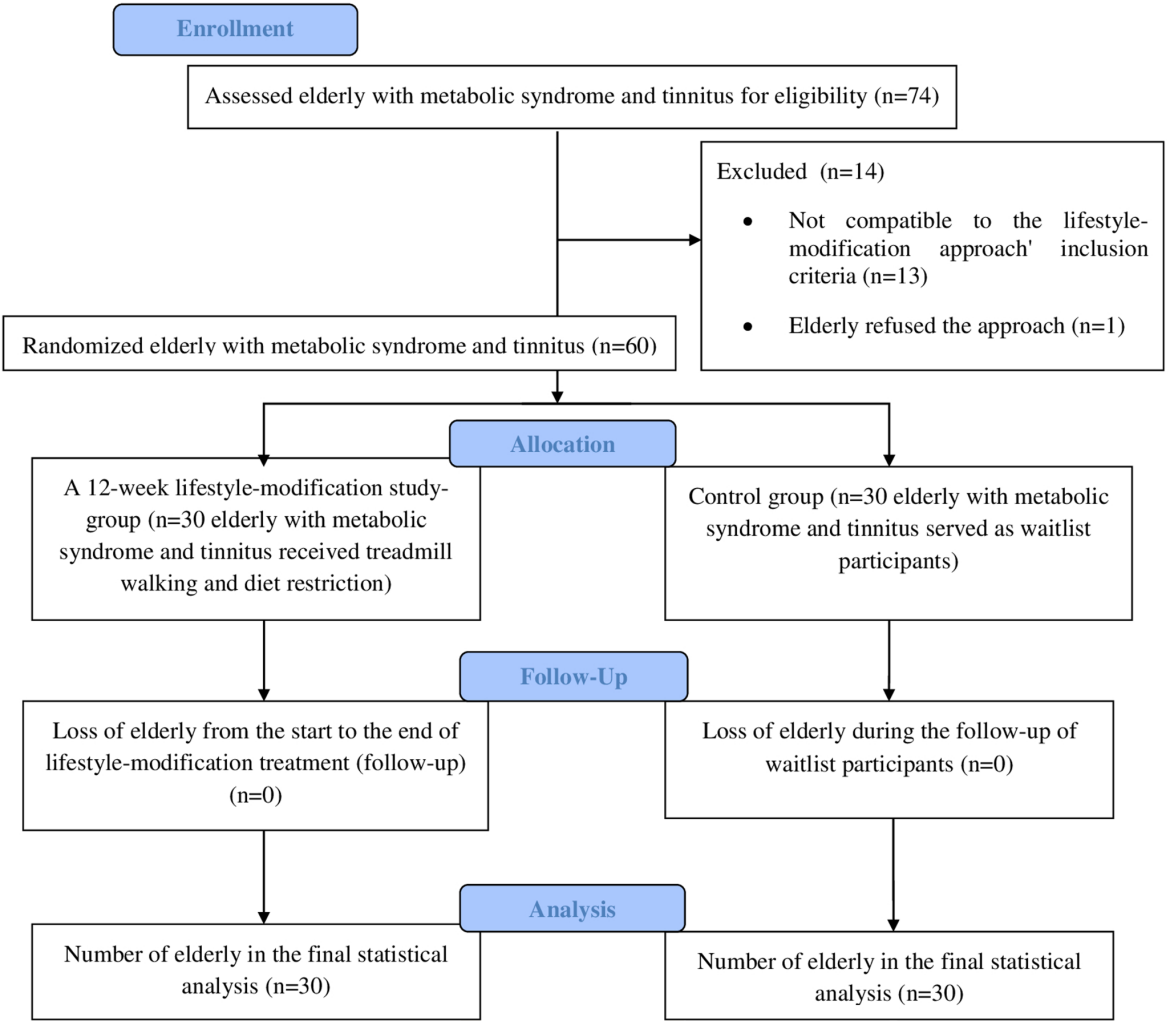
Flow chart of the sixty elderly with metabolic syndrome and tinnitus during the trail.

Metabolic-syndrome elderly with CSTC were randomly assigned to the trial’s study or control groups. The study group (*n* = 30 MS elderly with CSTC) received a lifestyle-modification approach, a program of diet restriction and treadmill walking exercise for 12 weeks). The MS elderly with CSTC in the control group were waitlist participants (*n* = 30) (Fig. 1). Randomization of MS elderly with CSTC was done via the closed envelope allocation. The person (a physiotherapy graduate) who performed the allocation of MS elderly with CSTC was not informed of the nature of the intervention.

### Lifestyle-modification approach’s components in MS elderly with CSTC

#### A) Diet-restriction component

Older participants with MS and CSTC followed the diet-restriction component of the approach (the CSTC participants’ diet was limited to 20–30% fatty components, 10–15% protein components, and 55–65% carbohydrate components). About 500 kilocalories per day were subtracted from CSTC participants’ basal metabolic rate (BMR) to manage the diet restriction component of the approach [16].

BMR of MS men with CSTC was managed/calculated from the Harris-Benedict equation/formula. BMR of CSTC men = 66.4730 + 13.7516 x CSTS men’s body mass in kilograms + 5.0033 x CSTC men’s height in centimeters– 6.7550 x CSTC men’s age in years [17].

BMR of MS women with CSTC was managed/calculated from the Harris-Benedict equation/formula. BMR of CSTC women = 655.0955 + 9.5634 x CSTS women’s body mass in kilograms + 1.8496 x CSTC women’s height in centimeters– 4.6756 x CSTC women’s age in years [17]. To track the CSTC elderly’s adherence to the diet-restriction component of the approach, a weekly face-to-face pre-scheduled appointment/interview was performed.

#### B) exercise component

A forty-minute on-treadmill (electric treadmill) walking class (3 classes per week) for seniors with MS and CSTC was provided. The walking class was broken down into sections for all of the obese elderly with MS and CSTC. Prior to and following CSTC seniors’ primary physical exertion section (this main CSTC seniors’ exercise stage was a 0.5-hour walking on the trial’s electric treadmill at 60–70% of CSTC seniors’ maximum heart rate), walking at 40–50% of CSTC seniors’ maximal heart rate was performed. It is performed before (as a warming section) and after (as a cooling section) CSTC seniors’ primary exercise Section. [7].

### Primary outcome *(CSTC severity)*

A 10-cm visual analogue scale (VAS) was used to assess MS seniors’ primary outcome of this trial, the severity of CSTC. The start of VAS denoted no severity of CSTC and the end of VAS denoted maximal severity of CSTC.

### Secondary outcomes

#### CSTC-related discomfort

Also, MS seniors’ CSTC-related discomfort was assessed using VAS. The start of VAS denoted no CSTC-related discomfort and the end of VAS denoted maximal CSTC-related discomfort.

#### Tinnitus handicap inventory

CSTC-related QoL in MS elderly was evaluated via tinnitus handicap inventory (THI). It is a 25-item QoL-assessing questionnaire in CSTC. In this lifestyle-modification trial, when a drop in MS elderly’s total THI score occurred, an improvement in CSTC-related QoL was considered [1, 12].

#### Body mass index

BMI was calculated by dividing MS older adult’s weight in Kg on MS older adult’s squared height in meters.

#### Components/variables of MS

CSTC seniors’ manually-measured blood pressure, CSTC seniors’ WC (an elastic tape was used to evaluate it), CSTC seniors’ FGIS (assessed with a glucose meter), and CSTC seniors’ serum TriGly and HDLipo (assessed with a blood analyzer) were assessed as components/variables of older participants’ MS.

### Blinding

Assessors of MS seniors’ BMI, VAS of CSTC severity, VAS of CSTC-related discomfort, components of MS, and THI were blinded to the designed lifestyle-modification approach.

### Power analysis of MS elderly with CSTC

The effect size of VAS of CSTC severity was 0.82. This effect size was a result of 16 pilot-trial elderly with MS and CSTC. Fifty persons with MS and CSTC were required as recommendations of this pilot trial, but 10 elderly were added. The power of this test was calculated at 80%.

### Statistical analysis

To be noted, all data of MS seniors with CSTC were normally distributed (the Smirnov test confirmed this notice in this lifestyle-modification trial). Pre-interventional statistical differences in MS seniors’ age and CSTC duration between CSTC groups were assessed via the unpaired test. The pre-interventional statistical difference of MS seniors’ sex between CSTC groups was assessed via the Chi-square test. Within or between groups of MS and CSTC, the statistical difference of MS seniors’ BMI, VAS of CSTC severity, VAS of CSTC-related discomfort, components of MS, and THI was assessed via repeated measure ANOVA. In this lifestyle-modification approach conducted on MS elderly with CSTC, the P value’s significance was < 0.05.

## Results

The between-group non-significant differences in MS seniors’ WC, MS seniors’ sex distribution/difference, MS seniors’ CSTC duration, MS seniors’ age, and MS seniors’ BMI are shown in Table 1 before starting the lifestyle-modification approach in MS elderly with CSTC.

**Table 1:** The based data of MeTS elderly with tinnitus complaint (Mean ± standard deviation)

The before-lifestyle modification data	Lifestyle changes group	Non-lifestyle changes group	*P* value
Age of MeTS elderly with tinnitus complaint [year]	70.03 ± 3.01	71.03 ± 4.04	0.281
Tinnitus duration in MeTS elderly [months]	42.70 ± 4.08	43.63 ± 4.24	0.392
Body mass index of MeTS elderly with tinnitus complaint [kg/m^2^]	32.91 ± 2.23	32.72 ± 1.67	0.710
Waist circumference of MeTS elderly with tinnitus complaint [cm]	110.16 ± 12.98	114 ± 10.62	0.216
MeTS elderly’s sex (percentage of females)	66.66	60	

The between-group before-lifestyle modification data are non-significant; MeTS: metabolic syndrome

The between-group non-significant difference in TriGly, VAS of CSTC severity, MS seniors’ blood pressure, VAS of CSTC-related discomfort, HDLipo, MS seniors’ THI, and FGIS is shown in Table 2 before starting the lifestyle-modification approach in MS elderly with CSTC.

**Table 2:** Outcomes of elderly’s MeTS and tinnitus complaint variables (Mean ± standard deviation)

Elderly’s MeTS and tinnitus complaint variables		Lifestyle changes group	Non-lifestyle changes group	Between groups’ *P* value
MeTS elderly’s Body mass index (Kg/m^2^)	Pre-lifestyle modification	32.91 ± 2.23	32.72 ± 1.67	0.710
	Post-lifestyle modification	30.76 ± 2.03	32.79 ± 1.87	< 0.001^*^
	P-value (within groups of MeTS elderly with tinnitus complaints)	< 0.001^*^	0.703	
MeTS elderly’s waist circumference (cm)	Pre-lifestyle modification	110.16 ± 12.98	114 ± 10.62	0.216
	Post-lifestyle modification	102.26 ± 14.46	114.80 ± 10.04	< 0.001^*^
	P-value (within groups of MeTS elderly with tinnitus complaints)	< 0.001^*^	0.154	
MeTS elderly’s systolic blood pressure (mmHg)	Pre-lifestyle modification	130.83 ± 6.36	132.90 ± 16.88	0.533
	Post-lifestyle modification	126.63 ± 7.23	133.60 ± 14.72	0.024^*^
	P-value (within groups of MeTS elderly with tinnitus complaints)	< 0.001^*^	0.439	
MeTS elderly’s diastolic blood pressure (mmHg)	Pre-lifestyle modification	82.36 ± 9.47	85.36 ± 11.48	0.274
	Post-lifestyle modification	78.50 ± 8.73	85.90 ± 12.99	0.012^*^
	P-value (within groups of MeTS elderly with tinnitus complaints)	0.001^*^	0.638	
MeTS elderly’s triglycerides (mg/dl)	Pre-lifestyle modification	196.50 ± 94.97	199.63 ± 49.87	0.809
	Post-lifestyle modification	166.66 ± 28.52	200.56 ± 51.02	0.002^*^
	P-value (within groups of MeTS elderly with tinnitus complaints)	< 0.001^*^	0.798	
MeTS elderly’s fasting blood glucose (mg/dl)	Pre-lifestyle modification	113.40 ± 11.87	116.23 ± 11.23	0.346
	Post-lifestyle modification	103.86 ± 10.68	116.93 ± 10.59	< 0.001^*^
	P-value (within groups of MeTS elderly with tinnitus complaints)	< 0.001^*^	0.477	
MeTS elderly’s high density lipoprotein_s_ (mg/dl)	Pre-lifestyle modification	45.13 ± 7.04	43.33 ± 7.44	0.340
	Post-lifestyle modification	49.16 ± 9.05	43 ± 7.03	0.005^*^
	P-value (within groups of MeTS elderly with tinnitus complaints)	< 0.001^*^	0.620	
MeTS elderly’s VAS tinnitus severity	Pre-lifestyle modification	6.90 ± 1.38	7.24 ± 1.43	0.359
	Post-lifestyle modification	4.74 ± 1.28	7.38 ± 1.49	< 0.001^*^
	P-value (within groups of MeTS elderly with tinnitus complaints)	< 0.001^*^	0.123	
MeTS elderly’s VAS tinnitus discomfort	Pre-lifestyle modification	6.98 ± 1.39	7.21 ± 1.17	0.492
	Post-lifestyle modification	4.08 ± 1.06	7.43 ± 1.16	< 0.001^*^
	P-value (within groups of MeTS elderly with tinnitus complaints)	< 0.001^*^	0.190	
MeTS elderly’s tinnitus handicap inventory	Pre-lifestyle modification	49.26 ± 9.93	52.66 ± 9.28	0.176
	Post-lifestyle modification	33.66 ± 9.09	53.06 ± 9.62	< 0.001^*^
	P-value (within groups of MeTS elderly with tinnitus complaints)	< 0.001^*^	0.607	

MeTS: metabolic syndrome; ^*^: significant P value; VAS: Visual analogue scale

While ANOVA reported no significant changes within the control group’s TriGly, BMI, VAS of CSTC severity, blood pressure, VAS of CSTC-related discomfort, HDLipo, THI, FGIS, and WC, these parameters showed significant improvements within the study group after completing the lifestyle-modification approach (Table 2).

With a superiority for the study group, after completing the lifestyle-modification approach in MS elderly with CSTC, post-interventional between-group comparison/matching showed significant improvements in all trial measures (TriGly, BMI, VAS of CSTC severity, blood pressure, VAS of CSTC-related discomfort, HDLipo, THI, FGIS, and WC) (Table 2).

## Discussion

In MS seniors with CSTC, besides improvements in BMI and components/variables of metabolic syndrome (HDLipo, FGIS, WC, TriGly, and blood pressure), the 12-week lifestyle-modification approach reduces THI, CSTC severity, and CSTC-related discomfort.

The mechanism explains the improvement in metabolic syndrome–associated THI, CSTC severity, and CSTC-related discomfort after a 12-week lifestyle modification approach (exercise with diet restriction) in obese MS elderly with CSTC is relatively unknown.

Tinnitus manifestations may occur from a rise in uncontrolled/spontaneous neuronal firing in the ear’s cochlea brought on by distress. Regular physical exertion has been proven to lower CSTC-associated symptoms of depression, anxiety, and QoL, which attenuates spontaneous in-cochlea neuronal firing [13].

According to the theory put forth by Loprinzi et al. [13] and Carpenter-Thompson et al. [18], physical exertion causes an increase in blood flow to the ear’s cochlea, which leads to an improvement in CSTC.

During exercise elevated heart rates may be associated with symptomatic relief of CSTC as a result of an increase in exercised individuals’ cardiac output (CO) and stroke volume (SV). Elevated cardiac distention, as a result of increased CO and SV, inhibits efferent sympathetic activity to exercised individuals’ ears [19]. This theory may explain the improved noise, ringing, and buzzing in the ears of our metabolic syndrome patients.

Regular physical exertion may help diabetic patients’ hearing functions last longer. Exercise-preventing or attenuating diabetic complications (increased platelet aggregation and blood viscosity, cochlear microangiopathy, ischemic cochlear injury, elevated cerebrospinal fluid’s blood glucose, auditory neuropathic changes in the central nervous system, and DM-induced encephalopathy) may prevent deterioration of hearing functions, lower inner-ear damage, and decrease the perception of loud sounds heard by tinnitus patients [11].

Regular physical exertion is linked to enhanced vascular/endothelial structure and functions, lower overall/systemic inflammation, decreased blood flow resistance, and attenuated blood pressure, all of which may, in theory, have an impact on DM-associated cochlear microangiopathy. Improvement of DM-associated cochlear microangiopathy could enhance CSTC. Also, physical exertion-induced increase in neurotrophic factor expression may help to attenuate aging- or DM-induced auditory neuropathy, hence CSTC improves [11].

Supporting us, studies reported that tinnitus complaints are low in physically active persons than in sedentary ones [13, 18, 20]. Also, the results of an online survey– conducted in 2015 - supported our results because this survey reported that physically active adults have good scores of tinnitus-related QoL and tinnitus severity than sedentary ones [18]. Also, old evidence suggested a strong role of exercise in protecting/preserving the functions of the central auditory system because physical aerobic exertion/training for 8 weeks enhances the exercising individuals’ auditory ability to recover from noise-induced auditory fatigue [21]. Consistent with our research, persons with DM who engage in physical exertion have better hearing functions than those who do not [11]. Also, headache - as a tinnitus-related symptom may increase in the absence of regular physical exertion among the elderly with tinnitus [22].

Again, body exercises (executed as part of a 3-month yoga intervention in CSTC patients) significantly improved THI and CSTC severity [23]. Also, CSTC-related QoL improved after body exercises (performed as a part of a 3-month yoga intervention) in CSTC patients [24].

Regarding weight loss, a study found tinnitus complaints’ prevalence in obese persons is 2.14 times higher than in those with normal BMI (i.e. BMI < 30 kg/m^2^) [8]. Accordingly, a reduction in CSTC patients’ excess body weight is expected to reduce CSTC symptoms [14, 15]. Because, after weight loss programs, OS and free radicals in the obese individuals’ circulatory system decrease [25]. Decreased OS is also known to improve CSTC symptoms [26].

Central obesity-associated increase in abdominal adipose tissue elevates levels of lipid peroxidation and fatty-acid release in obese persons’ circulatory systems evokes CSTC by inducing inner ear damage/dysfunction. In this direction, in obese patients with CSTC, lowering abdominal obesity, BMI, and lipids are associated with improved CSTC severity and CSTC-related QoL [12]. Also, in CSTC patients, reducing abdominal/central obesity after a lifestyle-modification approach (diet restriction and physical exertion) is associated with improved QoL and low complaints of tinnitus severity and discomfort [14, 15].

Parallel to our results in MS seniors with CSTC, the combined approach of diet restriction and physical exertion for 12 weeks in obese CSTC patients significantly improve their CSTC-related discomfort, BMI, CSTC severity, WC, and THI [14, 15]. Also, low-fat and low-refined-carbohydrate diets are protective against CSTC symptoms [14, 15, 27].

In CSTC patients with hyperlipidemia, the follow-up of a 24-month low cholesterol/fat diet with anti-hyperlipidemic drugs significantly improves CSTC patients’ TriGly, HDLipo, and tinnitus severity [28]. Also, studies reported that the risk and symptoms of tinnitus complaints are evoked by a diet-containing high levels of carbohydrates and fats [29, 30].

Supporting us, hyperinsulinemia patients who followed a 4-month supervised diabetic diet showed a significant decrease in hyperinsulinemia-associated CSTC severity [31]. Again, in line with the findings of the described lifestyle-modification trial’s results, hyperinsulinemic individuals who adhered to a dietary intervention were five times more likely to see improvements in their tinnitus complaints/symptoms than those who did not [32]. Also, the nutritional program containing low-carbohydrate and low-glycemic-index diets showed significant improvement in tinnitus severity and THI in tinnitus patients with metabolic disorders (IR, high levels of TriGly, and high levels of cholesterol) [33].

### Limitations

The results (BMI, CSTC severity, components of MS, THI, and CSTC-related discomfort) were not tracked after stopping the trial’s lifestyle-modification approach, so future CSTC trials in MS elderly must manage this limitation.

## Conclusion

In conclusion, BMI, CSTC severity, components of MS (WC, FGIS, blood pressure, TriGly, and HDLipo), THI, and CSTC-related discomfort showed significant improvements in response to the lifestyle-modification approach in MS elderly with CSTC.

## Data Availability

BMI, CSTC severity, components of MS (WC, FGIS, blood pressure, TriGly, and HDLipo), THI, and CSTC-related discomfort will be available on request.

## References

[1] Ismail AMA, Ali SM, Ghuiba K, Elfahl AMA, Tolba AMN, Ghaleb HAM (2022) Autonomic functions, tinnitus annoyance and loudness, and quality of life: Randomized-controlled responses to bee-humming (vibrational) respiratory training in tinnitus elderly. Complement Ther Clin Pract 48:101611. 10.1016/j.ctcp.2022.101611 35675742 10.1016/j.ctcp.2022.101611

[2] Ismail AMA, Aly MIE, Elfahl AMA (2022) Effect of acupuncture on tinnitus severity index in the elderly with non-pulsating tinnitus. Physiother Quarterl 30(1):57–60. 10.5114/pq.2021.108662

[3] Gaspar L, Makovnik M, Bendzala M, Hlinstakova S, Ocadlik I, Gasparov E (2011) Components of metabolic syndrome and their relation to tinnitus. In: Bahmad F Jr (ed) Up to date on tinnitus. InTech, Rijeka, pp 117–134

[4] Gibrin PC, Melo JJ, Marchiori LL (2013) Prevalence of tinnitus complaints and probable association with hearing loss, diabetes mellitus and hypertension in elderly. CoDAS 25(2):176–180. 10.1590/s2317-17822013000200014 24408248 10.1590/s2317-17822013000200014

[5] Ismail AMA (2023) Metabolic syndrome components response to the conducted 16-week randomised-controlled training trial on an elliptical trainer. Eur J Physiother 25(3):147–153. 10.1080/21679169.2021.2022756

[6] Ismail AMA (2023) Chat GPT in tailoring individualized Lifestyle-Modification programs in metabolic syndrome: potentials and difficulties?? Ann Biomed Eng 51(12):2634–2635. 10.1007/s10439-023-03279-x 37332005 10.1007/s10439-023-03279-x

[7] Ismail AMA, Hamed DE (2024) Erectile dysfunction and metabolic syndrome components in obese men with psoriasis: response to a 12-week randomized controlled lifestyle modification program (exercise with diet restriction). Ir J Med Sci 193(1):523–529. 10.1007/s11845-023-03412-8 37258850 10.1007/s11845-023-03412-8 PMC10808673

[8] Gallus S, Lugo A, Garavello W, Bosetti C, Santoro E, Colombo P, Perin P, La Vecchia C, Langguth B (2015) Prevalence and determinants of tinnitus in the Italian adult population. Neuroepidemiology 45(1):12–19. 10.1159/000431376 26182874 10.1159/000431376

[9] Petridou AI, Zagora ET, Petridis P, Korres GS, Gazouli M, Xenelis I, Kyrodimos E, Kontothanasi G, Kaliora AC (2019) The Effect of Antioxidant Supplementation in Patients with Tinnitus and Normal Hearing or Hearing Loss: A Randomized, Double-Blind, Placebo Controlled Trial. Nutrients. 2019 11(12):3037. 10.3390/nu11123037 10.3390/nu11123037 PMC6950042 31842394

[10] Yang P, Ma W, Zheng Y, Yang H, Lin H (2015) A systematic review and Meta-Analysis on the association between hypertension and tinnitus. Int J Hypertens 2015(583493). 10.1155/2015/583493 10.1155/2015/583493 PMC4735998 26881064

[11] Loprinzi PD, Gilham B, Cardinal BJ (2014) Association between accelerometer-assessed physical activity and objectively measured hearing sensitivity among U.S. Adults with diabetes. Res Q Exerc Sport 85(3):390–397. 10.1080/02701367.2014.930404 25141090 10.1080/02701367.2014.930404

[12] Ali Ismail AM (2023) mLipid profile response to acupuncture in obese patients with subjective tinnitus: a randomized controlled trial. J Acupunct Meridian Stud 16(1):11–19. 10.51507/j.jams.2023.16.1.11 36804817 10.51507/j.jams.2023.16.1.11

[13] Loprinzi PD, Lee H, Gilham B, Cardinal BJ (2013) Association between accelerometer-assessed physical activity and tinnitus, NHANES 2005–2006. Res Q Exerc Sport 84(2):177–185. 10.1080/02701367.2013.784840 23930543 10.1080/02701367.2013.784840

[14] Özbey-Yücel Ü, Aydoğan Z, Tokgoz-Yilmaz S, Uçar A, Ocak E, Beton S (2021) The effects of diet and physical activity induced weight loss on the severity of tinnitus and quality of life: A randomized controlled trial. Clin Nutr ESPEN 44:159–165. 10.1016/j.clnesp.2021.05.010 34330461 10.1016/j.clnesp.2021.05.010

[15] Özbey-Yücel Ü, Uçar A, Aydoğan Z, Tokgoz-Yilmaz S, Beton S (2023) The effects of dietary and physical activity interventions on tinnitus symptoms: an RCT. Auris Nasus Larynx 50(1):40–47. 10.1016/j.anl.2022.04.013 35568580 10.1016/j.anl.2022.04.013

[16] Ismail AMA, Saad AE, Draz RS (2023) Effect of low-calorie diet on psoriasis severity index, triglycerides, liver enzymes, and quality of life in psoriatic patients with non-alcoholic fatty liver disease. Reumatologia 61(2):116–122. 10.5114/reum/162995 37223373 10.5114/reum/162995 PMC10201385

[17] Luy SC, Dampil OA (2018) Comparison of the Harris-Benedict equation, bioelectrical impedance analysis, and indirect calorimetry for measurement of basal metabolic rate among adult obese Filipino patients with prediabetes or type 2 diabetes mellitus. J ASEAN Fed Endocr Soc 33(2):152–159. 10.15605/jafes.033.02.07 33442121 10.15605/jafes.033.02.07 PMC7784146

[18] Carpenter-Thompson JR, McAuley E, Husain FT (2015) Physical activity, tinnitus severity, and improved quality of life. Ear Hear 36(5):574–581. 10.1097/AUD.0000000000000169 25906172 10.1097/AUD.0000000000000169

[19] Pirodda A, Brandolini C, Ferri GG, Raimondi MC, Modugno GC, Esposti DD, Borghi C (2009) Possible influence on heart rate on tinnitus. Med Hypotheses 72(1):45–46. 10.1016/j.mehy.2008.09.006 18951725 10.1016/j.mehy.2008.09.006

[20] Chen S, Yang X, Jiang Y, Wu F, Li Y, Qiu J, Tong B, Liu Y (2023) Associations between physical activity, tinnitus, and tinnitus severity. Ear Hear 44(3):619–626. 10.1097/AUD.0000000000001306 36404413 10.1097/AUD.0000000000001306

[21] Ismail AH, Corrigan DL, MacLeod DF, Anderson VL, Kasten RN, Elliott PW (1973) Biophysiological and audiological variables in adults. Arch Otolaryngol 97(6):447–451. 10.1001/archotol.1973.00780010461003 4704439 10.1001/archotol.1973.00780010461003

[22] Bazoni JA, Dias ACM, Meneses-Barriviera CL, Marchiori LLM, Teixeira DC (2019) Possible association between the lack of regular physical activity with tinnitus and headache: Cross-sectional study. Int Arch Otorhinolaryngol 23(4):e375–e379. 10.1055/s-0039-1688469 31649754 10.1055/s-0039-1688469 PMC6805190

[23] Köksoy S, Eti CM, Karataş M, Vayisoglu Y (2018) The effects of yoga in patients suffering from subjective tinnitus. Int Arch Otorhinolaryngol 22(1):9–13. 10.1055/s-0037-1601415 29379573 10.1055/s-0037-1601415 PMC5786150

[24] Niedziałek I, Raj-Koziak D, Milner R, Wolak T, Ganc M, Wójcik J, Gos E, Skarżyński H, Skarżyński PH (2019) Effect of yoga training on the tinnitus induced distress. Complement Ther Clin Pract 36:7–11. 10.1016/j.ctcp.2019.04.003 31383447 10.1016/j.ctcp.2019.04.003

[25] Gao Z, Novick M, Muller MD, Williams RJ, Spilk S, Leuenberger UA, Sinoway LI (2013) Exercise and diet-induced weight loss attenuates oxidative stress related-coronary vasoconstriction in obese adolescents. Eur J Appl Physiol 113(2):519–528. 10.1007/s00421-012-2459-9 22814577 10.1007/s00421-012-2459-9 PMC3613987

[26] Ismail AM, El Melhat AM (2024) Erectile dysfunction in obese men with subjective tinnitus: a sedentary lifestyle as the link between the two problems that can be solved with exercise training. Health Probl Civiliz 18(3):257–258. 10.5114/hpc.2024.139096

[27] Gangemi SC (2010) DC D The dysglycemia test and its connection to temporomandibular joint dysfunction and tinnitus. Proceedings of the ICAK-USA

[28] Sutbas A, Yetiser S, Satar B, Akcam T, Karahatay S, Saglam K (2007) Low-cholesterol diet and antilipid therapy in managing tinnitus and hearing loss in patients with noise-induced hearing loss and hyperlipidemia. Int Tinnitus J 13(2):143–149 18229794

[29] Tomanic M, Belojevic G, Jovanovic A, Vasiljevic N, Davidovic D, Maksimovic K (2020) Dietary factors and tinnitus among adolescents. Nutrients 12(11):3291. 10.3390/nu12113291 33121120 10.3390/nu12113291 PMC7693091

[30] Dawes P, Cruickshanks KJ, Marsden A, Moore DR, Munro KJ (2020) Relationship between diet, tinnitus, and hearing difficulties. Ear Hear 41(2):289–299. 10.1097/AUD.0000000000000765 31356390 10.1097/AUD.0000000000000765 PMC7664714

[31] Basut O, Ozdilek T, Coşkun H, Erişen L, Tezel I, Onart S, Hizalan I (2003) Tinnituslu Hastalarda Hiperinsülinemi Sikliği ve Diyabet Diyetinin tinnitus Üzerine Etkisi [The incidence of hyperinsulinemia in patients with tinnitus and the effect of a diabetic diet on tinnitus]. Kulak Burun Bogaz Ihtis Derg 10(5):183–187 Turkish 12970590

[32] Lavinsky L, Oliveira MW, Bassanesi HJ, D’Avila C, Lavinsky M (2004) Hyperinsulinemia and tinnitus: a historical cohort. Int Tinnitus J 10(1):24–30 15379344

[33] Almeida TA, Samelli AG, Mecca Fdel N, De Martino E, Paulino AM (2009) Tinnitus sensation pre and post nutritional intervention in metabolic disorders. Pro Fono 21(4):291–297. 10.1590/s0104-56872009000400005 20098946 10.1590/s0104-56872009000400005

